# Apposition of *iroquois *expressing and non-expressing cells leads to cell sorting and fold formation in the *Drosophila *imaginal wing disc

**DOI:** 10.1186/1471-213X-7-106

**Published:** 2007-09-19

**Authors:** Eugenia Villa-Cuesta, Esther González-Pérez, Juan Modolell

**Affiliations:** 1 Centro de Biología Molecular Severo Ochoa, CSIC and UAM, Cantoblanco, 28049 Madrid, Spain; 2 Department of Ecology and Evolutionary Biology, Bow G-W, Brown University, Providence, RI 02912, USA

## Abstract

**Background:**

The organization of the different tissues of an animal requires mechanisms that regulate cell-cell adhesion to promote and maintain the physical separation of adjacent cell populations. In the *Drosophila *imaginal wing disc the *iroquois *homeobox genes are expressed in the notum anlage and contribute to the specification of notum identity. These genes are not expressed in the adjacent wing hinge territory. These territories are separated by an approximately straight boundary that in the mature disc is associated with an epithelial fold. The mechanism by which these two cell populations are kept separate is unclear.

**Results:**

Here we show that the Iro-C genes participate in keeping the notum and wing cell populations separate. Indeed, within the notum anlage, cells not expressing Iro-C tend to join together and sort out from their Iro-C expressing neighbours. We also show that apposition of Iro-C expressing and non-expressing cells induces invagination and apico-basal shortening of the Iro-C^- ^cells. This effect probably underlies formation of the fold that separates the notum and wing hinge territories. In addition, cells overexpressing a member of the Iro-C contact one another and become organized in a network of thin strings that surrounds and isolates large groups of non-overexpressing cells. The strings appear to exert a pulling force along their longitudinal axis.

**Conclusion:**

Apposition of cells expressing and non-expressing the Iro-C, as it occurs in the notum-wing hinge border of the *Drosophila *wing disc, influences cell behaviour. It leads to cell sorting, and cellular invagination and apical-basal shortening. These effects probably account for keeping the prospective notum and wing hinge cell populations separate and underlie epithelial fold formation. Cells that overexpress a member of the Iro-C and that confront non-expressing cells establish contacts between themselves and become organized in a network of thin strings. This is a complex and unique phenotype that might be important for the generation of a straight notum-wing hinge border.

## Background

Development of an organism requires that adjacent cell populations acquire different cell fates and give rise to different tissues. This is accomplished by the activation of specific sets of genes in each of the cell populations. In addition, cell segregation mechanisms have to be implemented to prevent cells from intermingling along boundaries between tissues and sometimes even within a tissue. In *Drosophila*, developmental boundaries were discovered in the wings, by means of genetically marked recombination clones, as straight lines that proliferating cells did not trespass [1] (reviewed by [2-5]). One of these boundaries corresponded to an invisible line that subdivided the wing into anterior (A) and posterior (P) compartments. Another boundary separated the dorsal (D) from the ventral (V) compartment, and corresponded to the wing margin. Subsequent work showed that the inheritable on or off state of the selector homeobox genes *engrailed *and *invected *(on in P and off in A) and *apterous *(on in D and off in V) defined the four compartments of the wing. Later work showed that short range cell-cell signalling occurred from P to A cells and between D and V cells. This was mediated by Hedgehog and Notch, respectively, and it was essential to maintain the A/P and D/V boundaries. Moreover, the short-range signalling gave rise to long-range signalling that organized the growth and patterning of the entire wing.

Differential cell-cell affinities were invoked to explain the presence of these compartment boundaries and their straight and smooth shape [6] (reviewed in [5]). Cells of a compartment would have an affinity for each other higher than for the cells of the neighbouring compartment. At the interface, cell-cell contacts would be minimized leading to straight and smooth boundaries. Aggregation experiments with mixtures of cells expressing either different amounts or different classes of cadherin adhesion molecules showed that cells could indeed sort out [7]. Moreover, overexpression of a single DE-cadherin in cell clones in the wing disc caused the clones to segregate from non-overexpressing cells and fuse together [8]. Thus, quantitative differences in the accumulation of adhesion molecules seem sufficient for cell segregation and may be critical for compartment boundaries to be formed and maintained. Still, the adhesion molecules involved in the compartment boundaries of *Drosophila *remain to be identified.

Cell lineage studies in the adult mesothorax suggested the presence of another cell restriction boundary between the notum and wing hinge [9]. However, the anatomical complexity of the wing hinge prevented its direct visualization by genetic marking in the adult fly. Moreover, analyses performed in the imaginal wing discs, the precursors of most of the mesothorax and the wings, indicated that the notum/wing hinge boundary is not a classical compartment boundary [10] (reviewed in [5]). Here, the notum region is defined by the expression of the homeodomain proteins of the *iroquois *complex (Iro-C, [11,12]), namely Araucan (Ara), Caupolican (Caup) and Mirror (Mirr), in the notum territory. The loss of these proteins from prospective notum cells transforms them into wing hinge cells [10]. Hence, the border of the Iro-C domain establishes the notum-wing hinge boundary. Mosaic analyses showed that during proliferation cells can trespass this boundary [10], which indicates that they can change the state of activation of Iro-C and consequently their notum or wing hinge identity. However, the change of identity is only possible during the second and early third instar. Later loss of Iro-C activity does not abolish notum commitment.

Differential cell adhesion is probably involved in formation and/or maintenance of the notum-wing hinge border. Indeed, dissociated notum and wing hinge cells sort out during aggregation [13]. Moreover, the borders of the Iro-C^- ^clones within the notum territory are smooth and rounded [10], which suggests that they tend to minimize contacts with the surrounding Iro-C^+ ^cells and posses a differential cell-cell affinity. However, this interpretation is complicated by the fact that the Iro-C^- ^clones are surrounded by a fold that spans several cell diameters and seems identical to that which separates the notum and wing hinge territories in the late third instar disc. Conceivably, formation of the fold might promote the roundness and smoothness of the clones.

Here we report that, within the notum anlage, cells not expressing Iro-C tend to join together and sort out from their Iro-C expressing neighbours. This supports a role for differential cell adhesion in formation and/or maintenance of the notum-wing hinge border. We also show that apposition of Iro-C expressing and non-expressing cells induces invagination and apico-basal shortening of the latter. This effect appears to be limited to a few cell diameters from the interface and probably underlies formation of the fold that separates the notum and wing hinge territories of the wing disc. In addition, we analyze in detail the previous finding [10] that cells overexpressing a member of the Iro-C arrange themselves in strings that can give rise to a bidimensional lattice that surrounds and isolates large groups of non-overexpressing cells. The data suggest that cells from different overexpressing clones have a tendency to establish contacts and exert a pulling force parallel to the direction of the strings.

## Results

### Cell clones lacking Iro-C tend to join together

**Figure 1:**
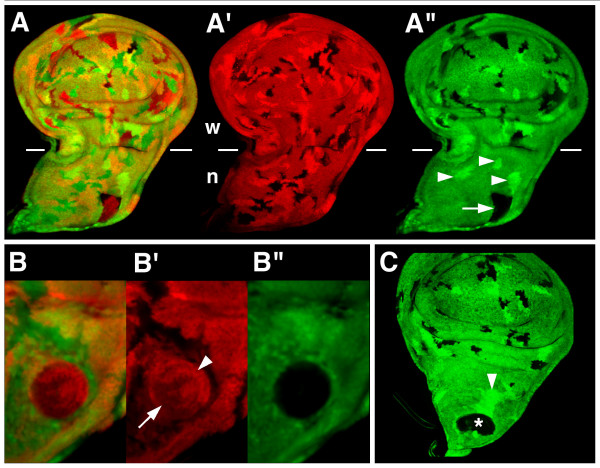
*iro*^*DFM3 *^clones within the notum territory can fuse together. Clones null for the Iro-C (*iro*^*DFM3*^) or lacking the *arm-lacZ *transgene were visualized by the absence of green or red, respectively. Homozygous *iro*^+/+ ^and *arm-lacZ*^+/+ ^twin clones were detected by the intense green and red signals, respectively. Both types of clones were induced simultaneously at 48 to 72 h AEL. (A-A") Imaginal wing disc displayed many *arm-lacZ*^- ^clones and twin spots in both the notum (n) and the wing and hinge regions (w). *iro*^*DFM3 *^and their corresponding twin spots were also quite numerous in the wing region, but only one *iro*^*DFM3 *^clone (arrow) and a few non-associated twin spots (arrowheads) were present in the notum. (B-B"). Close up of an *iro*^*DFM3 *^clone. Its cells are either *arm-lacZ*^+/- ^(arrow) or *arm-lacZ*^+/+ ^(arrowhead). (C) Wing disc displaying in the notum region a large *iro*^*DFM3 *^clone (asterisk, compare with clones in the wing and hinge region). The clone is associated with a large twin spot (arrowhead).

Third instar wing discs bearing mitotic recombination clones lacking the Iro-C (*iro*^*DFM3 *^clones) are noticeable in that they usually show only a single, relatively large clone within the notum territory, even when many clones are present in the hinge and wing regions (Figs. 1A", C; 43 out of 48 discs examined). Moreover, within the prospective notum territory, the *iro*^+/+ ^"twin spots" often lack their associated *iro*^*DFM3 *^clones (Fig. 1A"). Sometimes, a large twin spot is associated with the single, large *iro*^*DFM3 *^clone (Fig. 1C). We examined the reason for this scarcity of notum *iro *mutantclones by simultaneously inducing *iro*^*DFM3 *^clones and clones lacking the neutral marker *arm*-*lacZ*. The distribution of the latter showed that the frequency and size of the clones was similar in the notum and in the wing regions (Fig. 1A'). Hence, the scarcity of notum *iro*^*DFM3 *^clones could be due to either low viability, clone fusion, or both. Their relatively large size (Figs. 1A", C) suggested that fusion might indeed take place. This was evidenced by the recovery of *iro*^*DFM3 *^clones containing, for instance, *arm-lacZ*^+/- ^and *arm-lacZ*^+/+ ^cells (Figs. 1B–B"). Such clones should have originated from the fusion of *iro*^*DFM3 *^*arm-lacZ*^+/- ^and *iro*^*DFM3 *^*arm-lacZ*^+/+ ^independent clones. In the wing region, 18% of *iro*^*DFM3 *^clones (n = 64) and 15% of their twin clones (n = 59) comprised cells of two or more different lineages, as evidenced by their dosage of the *lacZ *marker. These mixed clones with composite lineages probably reflect chance encounters of initially separate clones. However, in the notum region, even though the density of neutral marker clones is similar to that in the wing region, the percentages were increased to 58% (*iro*^*DFM3 *^composite clones, n = 19) and 41% (twin composite clones, n = 24). We conclude that in a heterozygous Iro-C^+/- ^background, cells lacking Iro-C or containing two doses of the complex have a tendency to join together. Evidently, these observations do not rule out that low viability may also contribute to the scarcity of notum *iro*^*DFM3 *^clones.

### Apposition of Iro-C expressing and non-expressing cells induces invagination of the non-expressing cells

**Figure 2:**
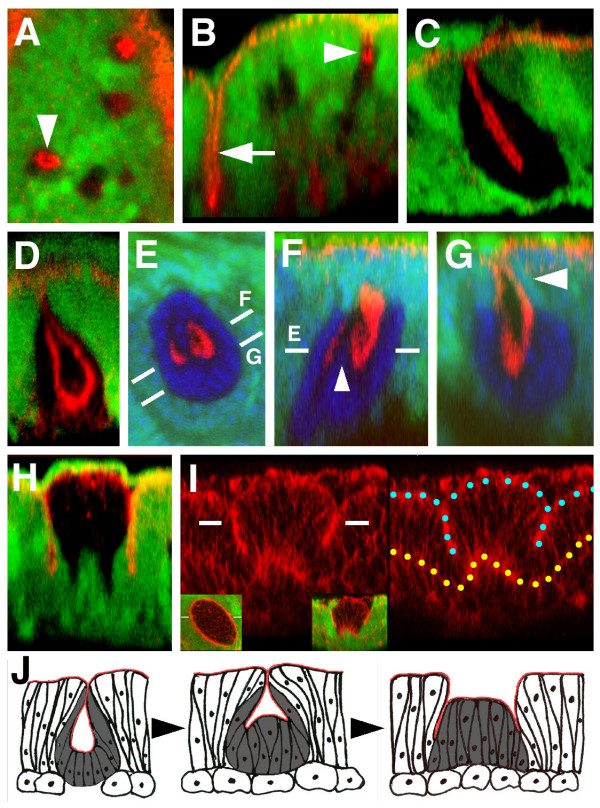
Morphology of *iro*^*DFM3 *^clones located within the notum territory. *iro*^*DFM3 *^cells: absence of green. (A-H) Ed apical marker, red. (E-G) TO-PRO-3 nuclear marker, blue. (A, B) Conventional and optical *z *section views respectively of *iro*^*DFM3 *^clones induced 72 to 96 h AEL. Arrowheads identify the same clone. Arrow: section through the notum/hinge fold. (C, D) Optical *z *section views of two different and larger *iro*^*DFM3 *^clones with the apical parts of their cells in aposed and separated configurations, respectively. (E-G)*xy *and two *z *section views, respectively, of a "W" shaped *iro*^*DFM3 *^clone. Dashes mark the approximate plane of the indicated views. Arrowhead: wild-type cells form the more apical region of the pouch. (H, I)*z *section views of two early induced (48–72h AEL and 24–48h AEL) clones stained for either Ed or Actin, respectively (red). Insets in I show the two channel *xy *(left) and *z *(right) views of the clone. Only the red channel is shown in the main panels. Blue and yellow dots mark the apical and basal regions, respectively, of the cells of the disc epithelium. (J) Suggested transition from pouch-shaped to cylindrical *iro*^*DFM3 *^clones that would occur during the development of the disc. *iro*^*DFM3 *^cells: grey. Apical regions: red. Round cells: adepithelial cells.

The notum *iro*^*DFM3 *^clones, induced between 48 and 72 hours AEL, undergo transformations towards a hinge fate [10]. To ascertain whether their roundish shape (as opposite to the wiggly, irregular contour of wild-type clones, Fig. 1A') and surrounding fold (Figs. 1B, C and 2H, I) are related to this transformation or to the apposition of cells expressing and non-expressing Iro-C, we examined *iro*^*DFM3 *^clones induced relatively late (72 to 96 h AEL) in the development of the disc. These clones do not transform towards wing hinge and give rise to either normal notum cuticle or invaginating vesicles of cuticle [10]. The clones, as observed in the notum region of the late third instar disc, were still roundish (Fig. 2A, E), but lacked a fold around them (Fig. 2B–D, G) and had a peculiar morphology. The apical regions of the mutant cells, as visualized in *z *optical sections by the adherens junction marker Echinoid (Ed, [14]), were recessed into the tissue (Fig. 2B, arrowhead). This invaginated apical region appeared to be mostly or exclusively formed by Iro-C mutant cells (Fig. 2A, B). This was clearer in larger, and presumably, older clones (Fig. 2C). Often, the *z *sections of these clones had the shape of a pouch, with the opposite apical regions of the cells not apposed to one another (Fig. 2D). The invaginations suggested that the mutant cells underwent an apical-basal shortening. This was clear in very small clones (Additional File 1A–C), which indicated that it was one of the earliest morphological changes underwent by their cells. The larger, pouch-shaped clones often sunk deeply into the adepithelial layer (Additional File 1D), although their cells never lost the continuity of their apical regions (Ed or Actin markers) with those of the adjacent non-mutant cells. In this large clones, it is uncertain whether cells at the clone interface also maintain the apico-basal shortening evident in cells located deeply into the pouch.

We reexamined in optical *z *sections the notum-to-hinge transformed *iro*^*DFM3 *^clones induced during the first/second instar. They were not pouch-shaped but cylindrical (Fig. 2H, I) and the fold surrounding them was formed by wild-type cells in the outer side and mostly, but not exclusively, *iro*^*DFM3 *^cells in the inner side. We asked whether this morphology, more complex than that of the clones induced at 72 to 96 h AEL, represented a more evolved stage of the *iro*^*DFM3 *^clones that could not be attained by the late-induced clones, possibly because cell proliferation did not last long enough. Some rare late-induced clones suggested that this might be the case (Fig. 2E–G). The apical zones of the cells deep in the pouch (Ed staining) of these clones were not disposed as in a concave bowl, but delineated a twisted form in a roughly "w" shape (Fig. 2F, arrowhead). This suggested that the cells at the bottom of the pouch might be recovering their normal apical-basal length, and consequently that, in older clones, the progeny of these cells might reach up to the apical plane of the epithelium (see model in Fig. 2J), producing an "eversion" of the clone. The lengthening would occur only in the internal cells of the clone, namely, those further removed from the border of the clone. Note also that the upper regions of the pouch of that rare clone were formed by wild-type cells (Fig. 2G, arrowhead). This could facilitate, upon eversion, formation of a fold with wild-type cells at the external side and mostly mutant cells at the internal side. According to this model, eversion of the clone would occur while the clone grew, although the rarity of the "w" stage suggests that eversion would be a relatively rapid process. In summary, the data above suggest that in the notum territory the apposition of Iro-C expressing and non-expressing cells causes the latter to undergo complex morphological changes. These would start with an apico-basal contraction and invagination of the mutant cells and, as the clone grew in size, would continue with a lengthening of the more internal cells of the clone (Fig. 2J). A fold at the interface between wild-type and mutant cells would be the end result of this process. The fold would probably help to smooth this interface.

Apical-basal cell shortening and invagination caused by apposition of Iro-C expressing and non-expressing cells probably induces formation of the fold between the notum and hinge territories during the third larval instar. This is supported by the observations that the fold arises at or very close to the border of the Iro-C expressing cells (Additional File 2A, B), the *z *sections of the fold in the late third instar disc (Additional File 2C) is most similar to those of the early-induced *iro*^*DFM3 *^clones (Fig. 2H, I), clones near the notum/hinge border can display folds continuous with that of the border [10], and removal of the Iro-C^+/- ^interface, removes the notum/hinge fold [15].

### *UAS-ara*-expressing cells from different clones establish connections

**Figure 3:**
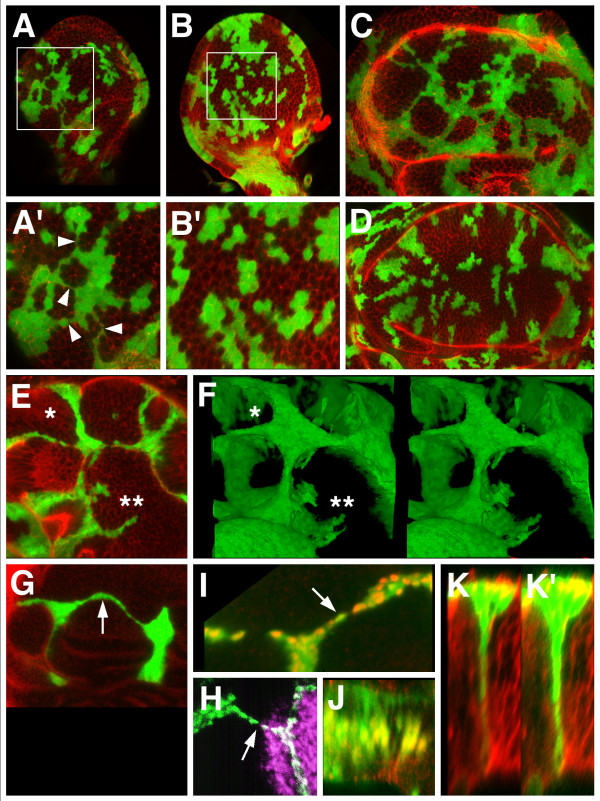
*UAS-ara*-expressing clones interconnect with one another. Clones (green) were detected by expression of *UAS-GFP*, except in H where anti Ara/Caup was used. Counterstaining (in red) was Actin (A-E, K, K'), anti RhoGEF2 (G), anti Ara/Caup (I, J) and (in purple) anti Engrailed (H). (A, B) Mid third instar wing discs with clones overexpressing *UAS-ara *and *UAS-GFP *or *UAS-GFP *alone, respectively, induced 48 hours earlier. Magnified views of the areas within squares are shown in A' and B'. Interconnections between the clones noticeable in A and A' (arrowheads) are absent in B, B'. In more mature third instar discs most clones overexpressing *UAS-ara *(C) are arranged in an interconnected net, but not so the clones not overexpressing the Iro-C gene (D). (E) *xy *confocal section of the wing pouch region of a disc bearing interconnected *UAS-ara *clones. (F) A stack of *xy *images of the same disc is shown in a stereo projection selecting only the *UAS-ara *expressing cells. Asterisks identify same areas in E and F. *UAS-ara *expressing cells tend to form "walls" separating large territories of non-expressing cells. In *x, y *sections these walls appear as thin strings. (G) A very long string (arrow) connecting two large separate clones. (H) An anterior compartment clone (green) and a posterior compartment clone (white) displaying connecting cells (arrow). Cell nuclei were stained with anti Ara/Caup antibody. Purple: Engrailed posterior marker. Picture modified from [16] (data courtesy of R. Diez del Corral). (I, J) *xy *and *z *optical sections, respectively, of a connecting string. Cell nuclei of overexpressing cells are shown in yellow or orange. Note that the string can be only one cell thick (arrow). (K, K') *z *optical section accross a single cell-wide wall. The green channel brightness has been strongly enhanced in K' to show the body of the cell(s) from apical to basal.

We further examined the behaviour of apposed cells containing different amounts of Iro-C products by overexpressing *araucan*, one of the Iro-C homeodomain genes [11], in cell clones. Previous work reported, but did not examine in detail, that in late third instar discs these clones appeared to contact each other and that their cells disposed themselves in thin, linear arrangements separating large, roundish islands of nonexpressing cells [10] (and Fig. 3C). We examined these clones in earlier discs and compared them with clones expressing only the neutral marker *UAS-GFP*. In young third instar discs, connections between clones were already apparent (Figs. 3A, A') and were most striking in older discs (Figs. 3C, E), where they interconnected the majority of clones. Although thin at some focal planes (Fig. 3A'), the connections contained nuclei at other planes (not shown; see also Fig. 3I). This indicated that they were composed of cells, rather than being cytoplasmic extensions. No connections were apparent between clones expressing only *UAS-GFP*, either in young (Fig. 3B, B') or late (Fig. 3D) third instar discs. Stereo views of stacks of confocal images clearly showed large masses of non-overexpressing cells arranged in "corrals" separated by thin walls of overexpressing cells (Fig. 3F). In *xy *sections the walls of the "corrals" showed as thin strings and we will refer to them as such. The strings could be just one cell thin (Fig. 3I–K) and quite long, spanning a relatively large fraction of the wing pouch width (Fig. 3G). As previously reported [16], strings could interconnect clones born in separate compartments, like the anterior and the posterior ones (Fig. 3H). This fact, together with the presence of thin, long strings among well separated mases of cells, suggests that the overexpressing cells had an extraordinary affinity for one another and reinforces the original suggestion that they were capable of joining together, even if born in different clones.

**Figure 4:**
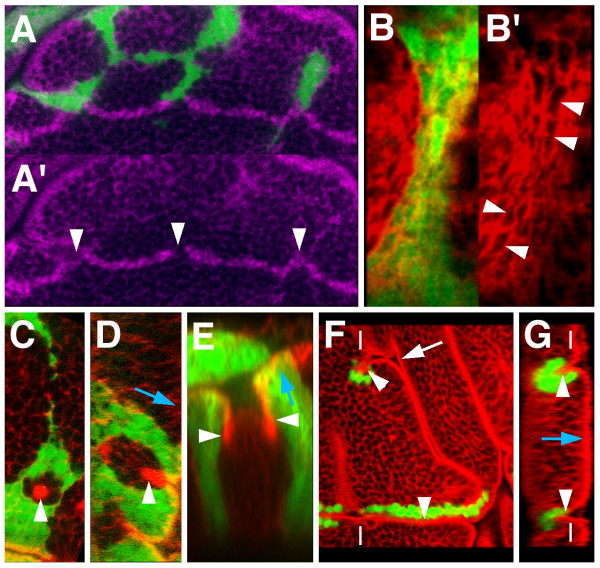
Some distinct features of *UAS-ara *overexpressing cells. *UAS-ara *expressing cells (green) were detected by coexpression of *UAS-GFP*, except in F and G where they were labelled with an anti Ara/Caup antibody. Counterstaining (in red) was Actin (B-D, F, G) or E-Cadherin (E). (A, A') *UAS-ara*-expressing cells distort the prospective wing margin (anti Cut staining, purple) by apparently pulling on it (arrowheads). (B, B') Apical *x, y *section of a connecting stripe between larger masses of UAS-ara expressing cells. Cell contours (arrowheads) are stretched parallel to the longitudinal axis of the stripe. (C, D) *xy *and *z *optical sections, respectively, of a small group of wild-type cells surrounded by *UAS-ara*-expressing cells. The former undergo apical-basal shortening (arrowheads). (E) *z *section of a group of wild-type cells surrounded by *UAS-ara *expressing cells. The apical marker DE-Cadherin shows that a fold has been formed at the interface (arrowheads). (F, G) *xy *and *z *optical sections, respectively, of *UAS-ara *expressing clones that have induced folds of the wing pouch epithelium (arrowheads) that comprise overexpressing and non-expressing cells. The folds can join with the extant folds of the disc (arrow). Dashes mark the approximate plane of the reciprocal view. For orientation, blue arrows in D, E and G point towards the apical part of cells.

The strings were either straight or smoothly curved around the masses of non-expressing cells. This suggested that they exerted a force in the longitudinal direction, like that of a thread wrapping around a stick. This interpretation was reinforced by the finding that the clones did pull on the dorsal/ventral compartment border, distorting it (Figs. 4A, A'), and that the contour of the cells of the strings, most easily observed in the apical sections, was stretched in a direction parallel to the length of the string (Figs. 4B, B'). Clones overexpressing *caupolican*, another member of the Iro-C, had the same morphology (not shown). Interconnecting clones were also observed in the eye-antenna disc (not shown). Flies with *UAS-ara *expressing clones did not survive to adulthood.

When the *UAS-ara *overexpressing cells surrounded a small number of wild-type cells, these could undergo an apical/basal shortening similar to those of the notum *iro*^*DFM3 *^cells surrounded by wild-type cells (Figs. 4C, D, compare with Figs. 1E, F). Groups of wild-type cells with a surrounding fold at the interface with the overexpressing cells, like that in between Iro-C^+ ^and Iro-C^- ^cells, were also observed (Fig. 4E). These results again suggested that the apposition of cells containing Iro-C proteins with those devoid of them (or with only low levels of them) caused the latter to undergo apical/basal contraction and the subsequent formation of a fold. Often, strings or zones harbouring many expressing cells were adjacent to a fold or even were associated with an ectopic fold that would join with an extant fold of the disc (Figs. 4F, G and [10]). Still, many *UAS-ara *expressing strings or groups of cells were not associated with folds, indicating that fold formation was not a necessary consequence of apposing Iro-C^+ ^and Iro-C^- ^cells and that it required additional contributing factors. A relatively sharp border of *UAS-ara*-expressing cells was important, since when expression was driven with a *Dpp-Gal4 *line, a fold formed only in the sharp border abutting the posterior compartment, but not in the more diffuse border within the anterior compartment [10].

## Discussion

Little is known of the molecules that prevent the mixing of cells between the subregions of the wing imaginal disc. Our data argue for a role of the Iro-C homeoproteins, which accumulate in the notum region of the disc, in promoting the differential affinity of notum and wing hinge cells. This differential affinity was discovered by the sorting out during aggregation of dissociated notum and wing hinge cells [13]. We have now shown that cell sorting behaviour indeed occurs in the intact wing disc: Iro-C^- ^cells located within the notum, which will develop as wing hinge cells, have a tendency to join together and minimize contacts with wild-type cells. Moreover, this behaviour seems to be induced by the different levels of Iro-C homeoproteins that occur in the apposing cells, rather than by the transformation of notum cells to hinge cells, since homozygous Iro-C^+ ^cells also tend to join together when located in an heterozygous Iro-C^+/- ^background of non-transformed cells. Moreover, wing hinge cells forced to express Iro-C by local activation of the EGFR pathway arrange temselves in roundish clones [17] suggesting a differential affinity with their non-expressing neighbours.

*UAS-ara *or *UAS-caup *overexpressing cells in a background of non-expressing cells also appear to contact one another. However, in this case the interaction between overexpressing cells is complicated by the fact that many of them arrange themselves in strings that can be as thin as a single cell and interconnect larger masses of overexpressing cells. The strings tend to surround areas with large numbers of cells not expressing Iro-C products or doing so at low levels. Conceivably, formation of the strings might be a passive process resulting from chance encounters by cells from different clones. If the overexpresing cells established strong adhesive interactions, the linkages between clones might be maintained and stretched into thin strings during disc growth. However, several observations support the alternative possibility that arrangements of the cells in strings is an active process. First, in young third instar discs bridges composed of a few cells arranged in chains that interconnect different overexpressing clones are quite abundant, while they are quite rare in control clones. Second, very long thin strings spanning many dozens of cell diameters can be observed in discs harbouring few clones. Third, pairs of clones born in the A and P compartments and whose main masses of cells are well separated from the compartment boundary can display interconnecting strings. Fourth, previous work [18] has shown that during the growth of the wing dics the cells of a clone normally remain together and usually separated from those of other clones. And fifth, control clones in the wing pouch grow mainly in a direction roughly perpendicular to the prospective wing margin [19] while strings between overexpressing clones take any possible direction. If indeed cells from different clones actively search for one another and establish contacts, the mechanisms involved are unknown. Other observations pertaining to clones overexpressing *UAS-ara*, like the distortion of the D/V boundary by clones contacting it (Fig. 4A, A'), the stretching of the cells in a direction parallel to the plane of the string (Fig. 4B. B'), and the relative roundness of the domains of wild-type cells surrounded by strings, suggest that the threads of overexpressing cells exert a pulling force along its longitudinal axis. This force could be actively generated by these cells or result from a restraining action of the strings on the growth of the encircled territories of nonexpressing cells. Regardless of the mechanism, it seems most likely that the overexpressing cells display a strong adhesion between themselves. Since clones of cells with a high differential affinity normally have roundish and smooth contours to minimize cell-cell contacts along the interface of the clone, it is of interest that the contour of the *UAS-ara *overexpressing clones, excepting for the stretched interconnecting strings, generally appear as wiggly as that of the wild type clones (Figs. 3A–D). This suggest the presence of a polarized affinity between the overexpressing cells that permits their arrangement in strings or threads a few cells thick, but does not tend to minimize their interface with non-overexpressing cells. To our knowledge, this phenotype of clones interconnected by strings is so far unique. It appears to be difficult to explain by simple differential affinity models.

We have shown that the apposition of cells expressing and not expressing Iro-C causes the non expressing cells to undergo apical-basal shortening and invagination. Our observations suggest that this effect has only a short range and that, as cells proliferate, those that are further removed from the interface recover a normal apical-basal length. This would provide a mechanism for the formation of the fold that surrounds the older clones or of that which separates the notum and wing domains of the imaginal disc. Since both these folds appear to be formed in an approximately symmetric way, at one side by Iro-C expressing cells and at the other by non-expressing cells, the apical-basal shortening effect may gradually and actively extend to the Iro-C expressing cells close to the interface. Alternatively, these may passively accommodate to the shortening of the non expresing cells. It should be stressed that previous evidence has already disclosed non-autonomous patterning effects of Iro-C^- ^clones located in the notum region on the surrounding Iro-C^+ ^cells [10]. Moreover, the suppression of the notum-wing hinge Iro-C border of expression negatively affects the growth of the wing disc [15]. Taken together, these lines of evidence suggest that the notum-wing hinge boundary is a source of signals that affect the growth and patterning of the surrounding tissue, an activity reminiscent of the signals that emerge from the apposition of cells at the A/P and D/V boundaries of the disc ([5], review).

The molecules responsible for the communication between Iro-C expressing and non-expressing cells are unknown. We have tested likely candidates, but the results have been negative. For instance, concerning fold formation, the role of the Rho GTPase pathway has been evaluated by producing *dRho*^*GEF*24.1 ^clones. *dRho*^*GEF*24.1 ^is activated by *folded gastrulation *and reiteratively required for epithelial folding and mesoderm invagination, but not for other processes regulated by Rho1 [20]. Contrary to previous evidence [20], these clones did not interfere in our hands with the notum/hinge fold, whose formation is dependent on the apposition of Iro-C expressing and non-expressing cells, or with the other extant folds of the disc. Overexpression of *UAS-Rho*^*GEF*2 ^in clones did induce apical-basal contraction of cells. However, these clones had low viability and the apical contraction might be due to a basal extrusion of the clones from the epithelium [21,22]. Overexpression of either *folded gastrulation *or a dominant negative form of Rho strongly disrupted the epithelium of the disc, so no conclusions could be reached. A change in Myosin II localization is needed for the apical constriction that precedes mesodermal invagination [23]. However, Myosin II accumulation was apparently unaffected in either Iro-C^- ^or *UAS-ara *overexpression clones (data not shown).

The arrangement of *UAS-ara *expressing cells in interconnecting clones was not disturbed by reduction of the MAP kinase pathway (coexpression with *UAS-raf*^*DN*^), which is active in the presumptive notum [17,24] and regulates cell adhesion [25], by the loss-of-function of DaPKC (coexpression with *UAS-DaPKC*^*DN*^), a protein required for apical/basal cell polarity [26], or by the reduction of function of Ephrin (coexpression with *UAS-DaEph*^*DN*^), a molecule involved in cell attraction/repulsion, adhesion/de-adhesion and migration in vertebrates (reviewed in [27]).

## Conclusion

The genes of the Iro-C, whose activity at the proximal part of the *Drosophila *wing disc is essential to define this territory as the notum anlage [10], also appear to participate in keeping the prospective notum cells separate from those of the adjacent wing hinge territory, which do not express the Iro-C genes. The available evidence suggests that the cell sorting behaviour between notum and hinge cells is induced by the different levels of Iro-C homeoproteins that occur in the apposing cells. Thus, the Iro-C homeoproteins appear instrumental in establishing not only the identity of the notum cells, but also the differential affinity of the notum versus the wing hinge cells, a property first recognized by the sorting behaviour during aggregation of dissociated notum and hinge cells [13].

These roles of the Iro-C seem similar to those of the homeobox selector genes *engrailed/invected *and *apterous *[2-5]. These genes are ultimately responsible, on the one hand, to impart "posterior" or "dorsal" identities to the cells of their respective expression compartments and, on the other hand, to promote the differential affinities that allow them to keep separate from the adjacent "anterior" or "ventral" cells. However, in contrast to these selector genes whose expression is inheritable maintained, this is not so for the expression of the Iro-C [10]. Hence, the border between the notum and winge hinge can be trespassed by cells when they change the state of Iro-C activation. Another difference between the borders at the anterior/posterior or dorsal/ventral compartments and at the notum/hinge territories is that only the last one is associated with a readily distinguishable morphological feature, namely, a fold of the epithelium. This fold appears to be induced by the apposition of the Iro-C expressing and non-expressing cells, which promotes an apical-basal shortening and invagination of the non-expressing cells. We propose a model that may explain how this change in cell configuration underlies formation of the notum/hinge epithelial fold.

Finally, we reexamine and extend the description of the unique and complex phenotype of disc cells that overexpress a member of the Iro-C [10]. The cells from different overexpressing clones establish contacts and become organized in a network composed of thin strings that surround and isolate large groups of non-overexpressing cells. The strings appear to exert a pulling force along their longitudinal dimension. Hitherto, we do not know of any similar phenotype.

## Methods

### Drosophila stocks

*Drosophila *stocks used were: *Df(3L)iro*^*DFM3 *^(= *iro*^*DFM3*^), *UAS-ara *[11]. Other stocks are described in FlyBase [28].

### Mosaic analyses

Mitotic recombination clones homozygous for *iro*^*DFM3 *^were induced by the FLP-FRT technique [29]. Flies *hsFLP1.22; mwh iro*^*DFM3 *^*FRT80B/TM6B *were crossed with *hsFLP1.22; ubi-GFP FRT80B *individuals. To obtain *iro*^*DFM3 *^mitotic recombination clones simultaneously with recombination clones for the *arm-lacZ *marker, *w hsFLP1.22; P{arm-lacZ} FRT40A/CyO*; *mwh Df(3L)iro*^*DFM3 *^*FRT80B/TM6B *flies were crossed with *FRT40A; ubi-GFP FRT80B *flies. Clones were induced by incubation of larvae at 37°C for 1 hour. Clones overexpressing *UAS-ara *were obtained by crossing flies carrying this transgene with *y w hs-FLP122; act-FRT y*^+ ^*FRT Gal4 UASGFP/Cyo *[30] flies. Clones were induced by incubation of larvae at 37°C for 15 minutes at 24–48 hours after egg laying (AEL) and 48–72 hours AEL.

### Antibody staining

Imaginal discs were fixed and stained as described [29]. Primary antibodies were: rat anti-Ara, which reacts with Ara and Caup proteins [10], guinea pig anti-Echinoid provided by Jui-Chou Hsu, rat anti-DE-Cadherin (DCAD2), rabbit anti-RhoGEF2 provided by Ronad D. Vale and mouse anti-Cut(2B10) from Developmental Studies Hybridoma Bank (DSHB), mouse anti-β-galactosidase from Promega, TO-PRO-3 from Invitrogen. Secondary antibodies were from Molecular probes. Rhodamine phalloidin was from Molecular Probes. Images were collected in an LSM510 META (Zeiss) confocal microscope.

## Competing interests

The author(s) declares that there are no comepeting interests.

## Authors' contributions

EVC and EGP carried out the experiments. EVC and JM designed the project and wrote the manuscript. All authors read and approved the final manuscript.

## Supplementary Material

Additional file 1 Apical-basal cell shortening and disposition within the notal epithelium of *iro*^*DFM3 *^clones induced 72 to 96 h AEL. *iro*^*DFM3 *^cells: absence of green. (A) Conventional *xy *view of two small *iro*^*DFM3 *^clones. Red: Actin staining. In clone b, the optical section shows only three nuclei (unstained material), but at least an additional one is visible in a more basal focal plane (not shown). (B, C) Optical *z *section views of clones b and c. Actin strongly labels the apical (arrowheads) and basal (asterisks) regions of the cells, and more weakly cell contours. Note that the apical regions of the cells of these small clones are already recesed into the epithelium (arrows), indicating an apical-basal shortening of the cells. Red chanels are also shown in white. (D) Optical *z *section view of a relatively large clone in a disc stained with anti Laminin alpha (Kumagai et al. 1997 FEBS Lett 412, 211–216) (red or white) and anti Integrin betaPS (DS Hybridoma Bank; Brower et al. 1984 Proc Nat Acad Sci USA 81, 7485–7489) (blue) antibodies. The extracellular matrix Laminin alpha delineates the invaginated apical region of the cells of the clone (arrowhead) and the continuous basal region of the cells (arrow). The integrin betaPS staining shows that the clone is deeply sunk into the adepithelial cell layer. e, epithelial cell layer; ad, adepithelial cell layer; yellow arrowhead, border between epithelial and adepithelial cells. Interestingly, levels of Integrin betaPS appear diminished in *iro*^*DFM3 *^cells. Click here for file

Additional file 2 An epithelial fold develops during the third instar developmental stage between the notum and wing hinge territories of the wing disc. The fold is located at the distal border of the Iro-C expression domain. Green: Actin; red: Ara/Caup. (A, B) Early and mid third instar wing discs showing the epithelial fold arising at the distal border of the *ara/caup *expressing domain (arrowheads). (C, D) Conventional and optical *z *section views, respectively, of the notum (n) – hinge (h) interface of a late third instar wing disc. *ara/caup*-expressing cells reach to the bottom of the fold (arrowhead). Dashes mark the approximate plane of the reciprocal view. Images A and B courtesy of Ruth Diez del Corral. Click here for file

## References

[B1] García-Bellido A, Ripoll P, Morata G (1973). Developmental compartmentalisation of the wing disc of *Drosophila*. Nature New Biol.

[B2] Dahmann C, Basler K (1999). Compartment boundaries: at the edge of development. Trends Genet.

[B3] Mann RS, Morata G (2000). The developmental and molecular biology of genes that subdivide the body of *Drosophila*. Annu Rev Cell Dev Biol.

[B4] Irvine KD, Rauskolb C (2001). Boundaries in development: formation and function. Annu Rev Cell Dev Biol.

[B5] Tepass U, Godt D, Winklbauer R (2002). Cell sorting in animal development: signalling and adhesive mechanisms in the formation of tissue boundaries. Current Op Genet Dev.

[B6] García-Bellido A, Santamaría P (1972). Developmental analysis of the wing disc in the mutant *engrailed *of *Drosophila melanogaster*. Genetics.

[B7] Steinberg MS, Takeichi M (1994). Experimental specification of cell sorting, tissue spreading, and specific spatial patterning by quantitative differences in cadherin expression. Proc Natl Acad Sci USA.

[B8] Dahmann C, Basler K (2000). Opposing transcriptional outputs of Hedgehog signaling and Engrailed control compartmental cell sorting at the *Drosophila *A/P boundary. Cell.

[B9] García-Bellido A, Morata G, Ripoll P (1976). Developmental compartmentalization in the dorsal mesothoracic disc of *Drosophila*. Dev Biol.

[B10] Diez del Corral R, Aroca P, Gómez-Skarmeta JL, Cavodeassi F, Modolell J (1999). The Iroquois homeodomain proteins are required to specify body wall identity in *Drosophila*. Genes Dev.

[B11] Gómez-Skarmeta JL, Diez del Corral R, de la Calle-Mustienes E, Ferrés-Marcó D, Modolell J (1996). *araucan *and *caupolican*, two members of the novel Iroquois complex, encode homeoproteins that control proneural and vein forming genes. Cell.

[B12] McNeill H, Yang CH, Brodsky M, Ungos J, Simon MA (1997). *mirror *encodes a novel PBX-class homeoprotein that functions in the definition of the dorso-ventral border of the *Drosophila eye*. Genes Dev.

[B13] Fausto-Sterling A, Hsieh L (1987). In vitro culture of Drosophila imaginal disc cells: aggregation, sorting out, and differentiative abilities. Dev Biol.

[B14] Wei SY, Escudero LM, Yu F, Chang LH, Chen LY, Ho YH, Lin CM, Chou CS, Chia W, Modolell J, Hsu JC (2005). Echinoid is a component of adherens junctions that cooperates with DE-Cadherin to mediate cell adhesion. Dev Cell.

[B15] Villa-Cuesta E, Modolell J (2005). Mutual repression between *msh *and Iro-C is an essential component of the boundary between body wall and wing in *Drosophila*. Development.

[B16] Cavodeassi F, Modolell J, Gómez-Skarmeta JL (2001). The Iroquois family of genes: from body building to neural patterning. Development.

[B17] Zecca M, Struhl G (2002). Control of growth and patterning of the *Drosophila *wing imaginal disc by EGFR-mediated signaling. Development.

[B18] González-Gaitán M, Capdevila MP, García-Bellido A (1994). Cell proliferation patterns in the wing imaginal disc of *Drosophila*. Mech Dev.

[B19] Baena-Lopez LA, Baonza A, Garcia-Bellido A (2005). The orientation of cell divisions determines the shape of *Drosophila *organs. Curr Biol.

[B20] Nikolaidou KK, Barrett K (2004). A Rho GTPase signaling pathway is used reiteratively in epithelial folding and potentially selects the outcome of Rho activation. Curr Biol.

[B21] Gibson MC, Perrimon N (2005). Extrusion and death of DPP/BMP-compromised epithelial cells in the developing *Drosophila *wing. Science.

[B22] Shen J, Dahmann C (2005). Extrusion of cells with inappropriate Dpp signaling from *Drosophila *wing disc epithelia. Science.

[B23] Dawes-Hoang RE, Parmar KM, Christiansen AE, Phelps CB, Brand AH, Wieschaus EF (2005). *folded gastrulation*, cell shape change and the control of myosin localization. Development.

[B24] Simcox AA, Grumbling G, Schnepp B, Bennington-Mathias C, Hersperger E, Shearn A (1996). Molecular, phenotypic, and expression analysis of vein, a gene required for growth of the *Drosophila *wing disc. Dev Biol.

[B25] Islam R, Kristiansen LV, Romani S, Garcia-Alonso L, Hortsch M (2004). Activation of EGF receptor kinase by L1-mediated homophilic cell interactions. Mol Biol Cell.

[B26] Sotillos S, Díaz-Meco MT, Caminero E, Moscat J, Campuzano S (2004). DaPKC-dependent phosphorylation of Crumbs is required for epithelial cell polarity in *Drosophila*. J Cell Biol.

[B27] Pasquale EB (2005). Eph receptor signalling casts a wide net on cell behaviour. Nat Rev Mol Cell Biol.

[B28] FlyBase. http://flybase.org.

[B29] Xu T, Rubin GM (1993). Analysis of genetic mosaics in developing and adult *Drosophila *tissues. Development.

[B30] Ito K, Awano W, Suzuki K, Hiromi Y, Yamamoto D (1997). The *Drosophila *mushroom body is a quadruple structure of clonal units each of which contains a virtually identical set of neurones and glial cells. Development.

