# Supplementary figures and images for: Apposition of iroquois expressing and non-expressing cells leads to cell sorting and fold formation in the Drosophila imaginal wing disc
Source: BMC Dev Biol. 2007 Sep 19;7:106. doi: 10.1186/1471-213X-7-106 (PMC2039746; doi:10.1186/1471-213X-7-106)

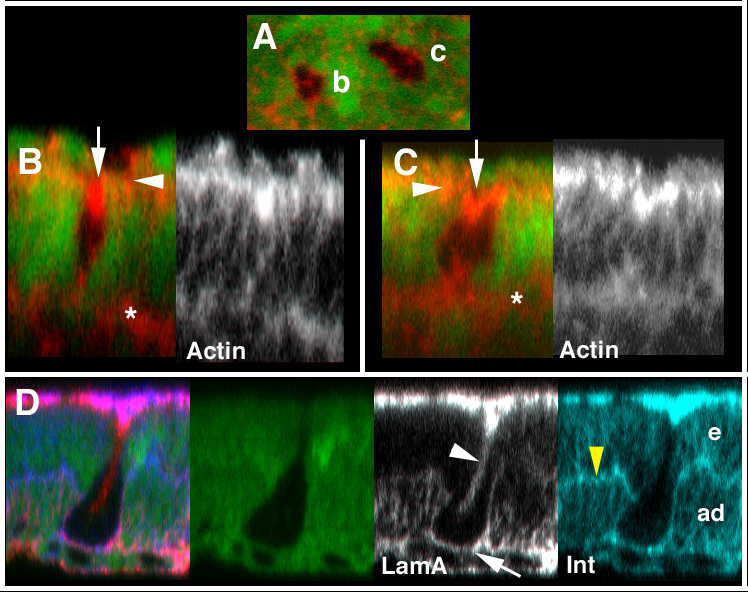

Supplement: Additional file 1 — Apical-basal cell shortening and disposition within the notal epithelium of iroDFM3 clones induced 72 to 96 h AEL. iroDFM3 cells: absence of green. (A) Conventional xy view of two small iroDFM3 clones. Red: Actin staining. In clone b, the optical section shows only three nuclei (unstained material), but at least an additional one is visible in a more basal focal plane (not shown). (B, C) Optical z section views of clones b and c. Actin strongly labels the apical (arrowheads) and basal (asterisks) regions of the cells, and more weakly cell contours. Note that the apical regions of the cells of these small clones are already recesed into the epithelium (arrows), indicating an apical-basal shortening of the cells. Red chanels are also shown in white. (D) Optical z section view of a relatively large clone in a disc stained with anti Laminin alpha (Kumagai et al. 1997 FEBS Lett 412, 211–216) (red or white) and anti Integrin betaPS (DS Hybridoma Bank; Brower et al. 1984 Proc Nat Acad Sci USA 81, 7485–7489) (blue) antibodies. The extracellular matrix Laminin alpha delineates the invaginated apical region of the cells of the clone (arrowhead) and the continuous basal region of the cells (arrow). The integrin betaPS staining shows that the clone is deeply sunk into the adepithelial cell layer. e, epithelial cell layer; ad, adepithelial cell layer; yellow arrowhead, border between epithelial and adepithelial cells. Interestingly, levels of Integrin betaPS appear diminished in iroDFM3 cells. [file 1471-213X-7-106-S1.jpeg]

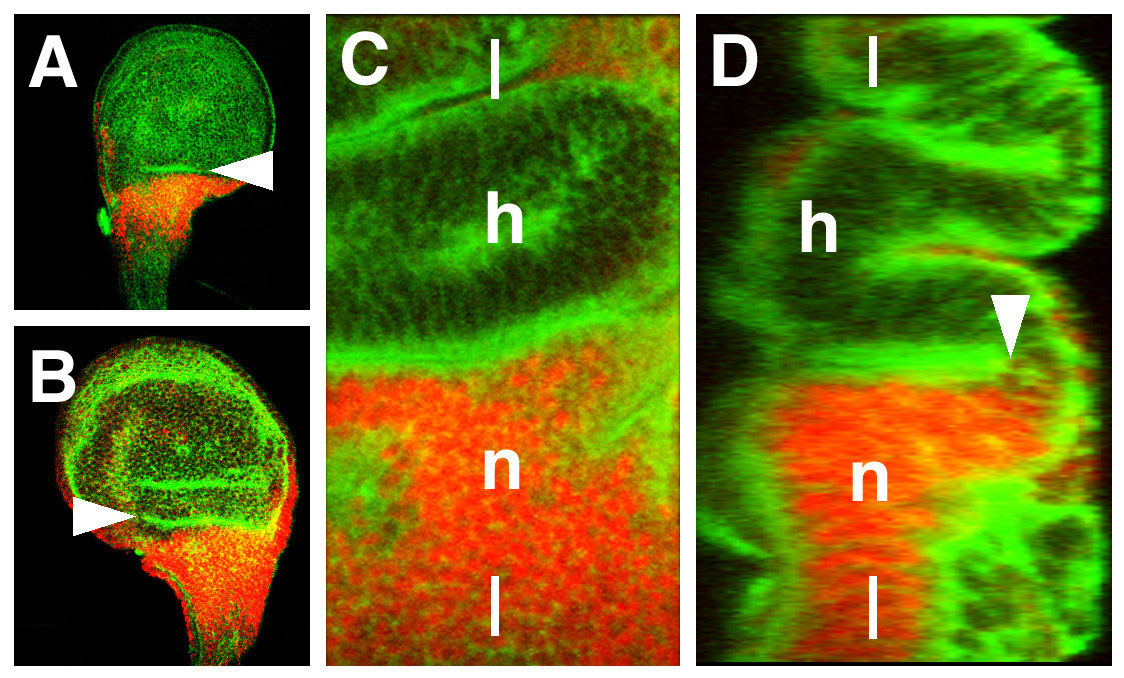

Supplement: Additional file 2 — An epithelial fold develops during the third instar developmental stage between the notum and wing hinge territories of the wing disc. The fold is located at the distal border of the Iro-C expression domain. Green: Actin; red: Ara/Caup. (A, B) Early and mid third instar wing discs showing the epithelial fold arising at the distal border of the ara/caup expressing domain (arrowheads). (C, D) Conventional and optical z section views, respectively, of the notum (n) – hinge (h) interface of a late third instar wing disc. ara/caup-expressing cells reach to the bottom of the fold (arrowhead). Dashes mark the approximate plane of the reciprocal view. Images A and B courtesy of Ruth Diez del Corral. [file 1471-213X-7-106-S2.jpeg]
